# Maternal satisfaction and associated factors towards institutional delivery service at public hospitals in the Somali region, Eastern Ethiopia

**DOI:** 10.1371/journal.pone.0277224

**Published:** 2022-11-09

**Authors:** Zemenu Shiferaw, Abdirahman Mahad, Semehal Haile

**Affiliations:** 1 Department of Reproductive Health and Population Studies, School of Public Health, College of Medicine and Health Science, Bahirdar University, Bahirdar, Ethiopia; 2 Department of Public Health, College of Medicine and Health Science, Jigjiga University, Jigjiga, Ethiopia; 3 Department of Midwifery, College of Medical and Health Sciences, Jigjiga University, Jigjiga, Ethiopia; Department of Medical Research, MYANMAR

## Abstract

**Background:**

Client satisfaction reflects the gap between the expected service and the experience of the service, from the client’s point of view. Maternal satisfaction with delivery services affects the selection of birthplaces and helps to identify gaps between actual health care and desired health care outcomes. There is a dearth of evidence on the factors of client satisfaction towards delivery services in the Somali region.

**Objective:**

The aim of this study was to assess maternal satisfaction and associated factors towards institutional delivery service at public hospitals in the Somali region, Eastern Ethiopia, in 2018.

**Methods:**

An institution-based cross-sectional study design was conducted from December 1–30, 2018, on a total of 320 delivering mothers who gave birth. A systematic random sampling technique was used. A pretested structured questionnaire was used for data collection through a face-to-face interview. The data was checked for completeness and entered into EpiData. Statistical analysis was done by SPSS version.20. Binary logistic regression was used to identify factors. Multivariable analysis was done to compute adjusted odds ratios at 95% CI and a P-value <0.05 was used to declare a statistically significant association.

**Results:**

The overall maternal satisfaction towards delivery service was 76.60% (95%CI: 71.90, 80.90). The short duration of labor below 12 hours (AOR = 1.80, 95%CI: 1.01, 3.2), waiting time below 15 minutes (AOR = 2.78, 95%CI: 1.10, 6.99) and planned pregnancy (AOR = 3.68, 95%CI: 1.59, 8.47) were significantly associated with mother’s satisfaction towards delivery services.

**Conclusions:**

This study revealed that more than three-quarters of mothers were satisfied with the institutional delivery service. Longer waiting time, suffering from persistent labor and having unplanned pregnancies hindering factors for maternal satisfaction. Therefore, programs on education and counseling towards pregnancy planning should be strengthened. Efforts should be made on availing adequate skilled birth attendants to manage persistent labor and handle longer waiting times.

## Introduction

Global maternal mortality declined by about 38% between 2000 and 2017. However, pregnancy and childbirth-related complications result in nearly three hundred thousand maternal deaths. Over eight hundred ten women die every day due to these complications [1]. Developing countries take a higher share (94%) of the global maternal deaths. Sub-Saharan Africa and Southern Asia account for 85% of the global burden of maternal death. Sub-Saharan Africa alone accounts for 56%, while Ethiopia accounts for 3% to 5% of global maternal deaths. A significant disparity was observed in the maternal mortality ratio (MMR) between the regions. The reported MMR in the least developed countries was 415 per 100,000 live births, while in Europe and Northern America it was 12 per 100,000 live births [1, 2].

Despite global and national commitments to reduce maternal deaths, a significant number of maternal deaths still occur. The maternal mortality ratio is set to be less than 70 per 100,000 live births in the Sustainable Development Goals (SDGs). It also aimed to reduce neonatal mortality to at least 12 per 1000 live births and under-five mortalities to at least 25 per 1000 live births by 2030 [3]. The accessibility of institutional delivery alone is not adequate for these goals; it is also the quality of care (including satisfaction) that saves the lives of mothers and newborns [4]. The Ethiopian Demographic Health Survey (EDHS) 2016 showed that institutional deliveries were 26% but 73% of mothers gave birth at home [4, 5].

The World Health Organization (WHO) recommended a skilled birth attendance for every birth to avert maternal deaths from preventable childbirth-related complications. The provision of satisfying institutional delivery service enhances service utilization According to the WHO recommendation, quality improvement and health care success involves routine assessment of women’s satisfaction [6, 7]. Maternal satisfaction with delivery care is critical to the health and well-being of both women and infants [8]. Satisfaction is the state of happiness or pleasure resulting from an action, incident, or service. It is decided mainly by clients’ expectations and experiences [9]. A satisfied user is highly likely to come back to the health facility in the future. They also advocate the service to other potential users including their neighbors and relatives [10]. Perceived quality is a key factor affecting service utilization. Hence, assessing women’s perception of care and satisfaction with services is important [5, 11].

According to the evidence from different countries, a disparity in the levels of maternal satisfaction with delivery care was observed. Studies from Dhaka, Bangladesh, South Australia, Kenya, and South Africa, have found that maternal satisfaction with delivery care ranged from 51.9% to 92.3% [11–15]. While in Ethiopia, different studies showed that overall maternal satisfaction was between the ranges of 19% to 79.1% [12–14].

Several factors were identified as predictors for the level of satisfaction with delivery care. These include women’s education, place of residence, women’s monthly income, cost of the service, convenience, and physical accessibility, and others [1, 11, 12–15]. In addition, perceived caregivers’ attitudes, and the interpersonal, and technical aspects of delivery care affect maternal satisfaction [6, 16, 17].

There is little evidence of maternal satisfaction with delivery services in Ethiopia’s pastoralist and agro-pastoralist regions, particularly in the Somali region. Therefore, this study was conducted to assess the level of maternal satisfaction and its associated factors towards institutional delivery services at the public hospitals in the Somali region, Eastern Ethiopia.

## Methods

### Study design, setting and period

This study was done in the Somali regional state, Eastern Ethiopia. An institution-based cross-sectional study design was conducted from December 1–30, 2018. It aimed to assess the maternal satisfaction and associated factors towards institutional delivery services at the public hospitals in the Somali regional state. The Somali regional state is one of the hard-to-reach pastoralist and agro-pastoralist areas in Ethiopia. The region remains in poor health status compared with the other regional states of the country. The number of comprehensive high-referral hospitals was not more than six. The utilization of basic maternal health services remained very low in the region. For instance, modern contraceptive use was not more than 1%, only 43.6% of pregnant women received antenatal care from skilled providers and only 17.9% of pregnant women gave birth at the health facilities [4, 18].

### Population

The source populations were all women who visited public hospitals in the Somali region for institutional delivery services (childbirth services). All randomly selected women who gave birth at public hospitals in the Somali region were the study population.

#### Inclusion criteria

All mothers who gave birth at the Karamara general hospital and Sheik Hassen Yabere memorial referral hospital (SHYMRH) within the study period were included.

#### Exclusion criteria

Those mothers who were unable to respond (e.g., who were unable to talk) or were very sick were excluded.

### Sample size

A single population proportion formula was used to determine the sample size. The assumptions were a 95% CI, a 5% margin of error, a 10% non-response rate, and a 74.6% prevalence of maternal satisfaction in the Oromia region, Ethiopia [11]. The final sample was found to be 320.

### Sampling procedure

In the Somali regional state, there are six higher referral hospitals that provide basic and comprehensive obstetrics and newborn care. Then, by lottery, Karamara general hospital and Sheik Hassen Yabere memorial referral hospital (SHYMRH) were chosen. The numbers of women who came for delivery service (childbirth) were estimated by reviewing the average last three-month delivery service report of the two hospitals. Accordingly, nine hundred women gave birth at SHYMR hospital and another five hundred gave birth at Karamara hospital in the last three months. Then, the sample was proportionally allocated to each hospital, i.e., 206 women were taken from SHYMR hospital and 114 women were taken from Karamara Hospital. Finally, the study participants were selected by systematic random sampling. The sampling interval (value of K) was four (every fourth woman was interviewed).

### Data collection tools

The data was collected by a pretested structured questionnaire developed from different literature. The data collection technique was interviewer-administered by trained data collectors. Four female clinical nurses who were fluent in Somali and English were recruited for data collection. The questionnaire had three parts: Socio-demographic and economic characteristics assessment part, the obstetric characteristics assessment part, and the satisfaction assessment part. The content validity of the tool was checked and a pretest was done on 5% of the sample size. An interviewer-administered interview was carried out in the postnatal unit. To avoid the social desirability bias, the data collectors were not employees of the hospitals, and the exit interview was done away from the delivery unit. To assure the data quality the collected questionnaires were reviewed and cross-checked for completeness, accuracy, and consistency by the supervisor and principal investigator, and corrective measures were taken. Incomplete questionnaires were re-administered accordingly. In addition, the questionnaire was firstly prepared in English and translated to Somali, and then translated back to English in order to ensure its consistency.

The satisfaction of the mothers was measured using questions that were adopted from the Donabedian quality assessment framework which is presented using a five-point Likert scale ranging from “very dissatisfied” to “dissatisfied, neutral “satisfied “and “very satisfied” [4, 19]. The overall satisfaction assessment questions were composed of two subsections. The first section was structure-related satisfaction assessment items which contain seven questions. The second subsection was for process-related satisfaction assessment which contains ten questions.

### Measurement

Maternal satisfaction: it is the satisfaction of the mothers towards delivery care/service. It is the care level obtained that increases the likelihood of future utilization of maternal health services [20].

Assessing the level of satisfaction: For overall satisfaction, a Five-Point-Likert scale was used: (1) very dissatisfied, (2) dissatisfied, (3) neutral, (4) satisfied, and (5) very satisfied. During the scoring of satisfaction, a score of “one” was given for very dissatisfied and dissatisfied, while a score of “zero” was given for neutral, satisfied, and very satisfied [4, 18, 20].

Overall Satisfaction: The overall satisfaction was computed from process-related satisfaction and structured-related satisfaction scored on a total of seventeen items. Structure-related satisfaction was computed from seven structure-related items, while process-related satisfaction was computed from ten process-related items.

- Satisfied:—Mothers who scored 75% or more on the items of the Patient Satisfaction Questionnaire were categorized as “satisfied” for the overall satisfaction level. Each response of ‘very satisfied’ and ‘satisfied’ was classified as satisfied [4, 19, 21].
- Unsatisfied:—mothers who scored below 75% on the items of the Patient Satisfaction Questionnaire, were categorized under “unsatisfied” for the overall satisfaction level. Each response of ‘very dissatisfied’, ‘dissatisfied’, and ‘neutral’ as unsatisfied [4, 19, 21].

A delivery service: is a service/care provided to a woman who comes to the health facility for childbirth by an accredited skilled provider such as nurses, medical doctors, midwives, or health officers during the recent birth. It involves the recording of the progress of labor, and maternal and fetal conditions using a Partograph [22].

### Data processing and analysis

The collected data was checked for completeness, and data entry was performed by Epi Data version 4.1. Then, it was exported to SPSS Windows Version 20 for analysis. Descriptive statistics were computed for each variable. Chi-square testing was used and normality was checked. A binary logistic regression analysis was used to identify factors associated with maternal satisfaction (the outcome variable). Firstly, a bivariate analysis was done between the factors and maternal satisfaction (the outcome variable). In the Bivariate analysis, those variables with a p-value of below 0.25 were considered to be candidates/covariates for the multivariable logistic regression analysis. A p-value of below 0.25 was used for covariate selection to ensure maximum prediction of the outcome variable. Secondly, the multivariable analysis was done and adjusted odds ratios were generated with p-values and 95% CI. Those factors which were associated with the outcome at a p-value less than 0.05 were identified as independent predictors of maternal satisfaction. Multi-collinearity was checked using the Variance Inflation Factor (VIF). Model goodness of fit was checked by the Hosmer-Lemeshow goodness-of-fit test.

### Ethical approval and consent to participate

A written ethical approval letter was taken from the Jigjiga University Research Ethics Review Committee (Ref.No RERC/027/2018). Each study participant was asked to sign a written consent before data collection. For those participants who were unable to read the consent, a verbal explanation was given to them and their consent was recorded by the data collectors. They were informed about the objective of the study, the confidentiality of their data, and the right to refuse participation. To maintain the auditory and visual privacy of the participants, the interview was carried out in a separate room by trained data collectors who were not affiliated with the facilities studied.

## Results

### Socio-demographic characteristics of the mothers

A total of 320 mothers were interviewed, yielding a 100% response rate. The mean age of the participants was 26.0 (±5.7) years. More than two-thirds (228) of the study participants were aged from 21 to 34 years. More than half (249) of the study participants were ethnic Somalis and 271(84.6%), were Muslims. The majority, 296 (92.5%) were married, while 208 (65%) were housewives. More than two-thirds, 258 (80.6%), of the respondents were urban residents. Of the total study participants, 167 (52.2%) had no formal education, and 152 (47.5%) mothers had an income of less than 3000 Ethiopian birrs (Table 1).

**Table 1:** Socio-demographic characteristics of mothers who gave birth at public hospitals in the Somali region, Eastern Ethiopia, 2018.

Variable (n = 320)	Category	Frequency	Percentage (%)
**Maternal age**	≤20	62	19.4
	21–34	228	71.3
	35–49	30	9.3
**Marital status**	Married	296	92.5
	Others*	24	7.5
**Religion**	Muslim	271	84.7
	Non-muslin	49	15.3
**Ethnicity of the mothers**	Somali	249	77.8
	Non-Somali	71	22.2
**Educational status**	No formal education	167	52.2
	Formal education	153	47.8
**Occupation of the mothers**	House wife	208	65.0
	Government employee	48	15.0
	Others**	64	20.0
**Residence**	Rural	62	19.4
	Urban	258	80.6
Average monthly income in birr ^a^	<3000	152	47.5
	3100–5000	99	30.9
	>5000	69	21.6

* widowed, divorced

**private employee, NGO, daily labor, and student

^a^1 USD were exchanged 28.45 birr in 2018.

### Obstetric history of the mothers

Of the total respondents, 200 (62.5%) of them had 2 to 5 children. One hundred thirty-eight (43.1%) of the study participants preferred to give birth at the health facility because others consulted them to do so. The majority 284 (88.8%) of the respondents replied that their current pregnancy was planned. One hundred ninety-five (60.9%) of respondents had labor that persisted below 12 hours. Regarding mode of delivery, three-quarters (75.9%) of the respondents had spontaneous vaginal delivery. Thirty-six (11.2%) of the study participants experienced complications after delivery. On the other hand, two hundred fifty-six (80%) of the study participants had normal fetal outcomes (Table 2).

**Table 2:** Obstetric history of mothers who gave birth at public hospitals in the Somali region, Eastern Ethiopia, 2018.

Variables	Category	Frequency	Percentage (%)
**Parity**	1	76	23.8
	2–5	200	62.5
	>5	44	13.7
**Reason to prefer hospitals to give birth**	Consulted by others	140	43.8
	Satisfied with previous birth	96	30.0
	Referred from other place	84	26.2
**Status of pregnancy**	Planned	284	88.8
	Unplanned	36	11.2
**Length of labor(in hours)**	≤12hours	195	60.9
	>12hours	125	39.1
**Mode of delivery**	SVD	243	75.9
	Caesarean section	37	11.6
	Instrumental delivery	40	12.5
**Maternal outcome** *	Normal	284	88.8
	Having complication	36	11.2
**Fetal condition** **	Alive	256	80.0
	Having complication	46	14.4
	died	18	5.6
**ANC follow up**	Yes	242	75.6
	No	78	24.4
**Number of ANC visit**	<4visit	147	60.5
	≥4visit	96	39.5
**Waiting time**	≤15minute	295	92.1
	>15minute	25	7.9

*A normal maternal outcome occurs when the mother has no complications after giving birth.

**A normal fetal outcome is one in which there is no birth defect, low birth weight, preterm birth, stillbirth, or other complications.

### Maternal satisfaction

Overall maternal satisfaction was found to be 76.6%. Structure-related satisfaction and process-related satisfaction were taken into consideration to compute overall satisfaction.

#### Structure- related satisfaction of the mother

The overall level of structured-related maternal satisfaction was 76.9%. The majority of the mothers, 271 (84.7%), were satisfied with the number of health care providers in the labor and delivery rooms, and 266 (83.1%) of them were satisfied with the availability and adequacy of medical supplies and drugs. Two hundred fifty-seven participants (80.3%) were satisfied with the sufficiency and cleanliness of the labor rooms, and 266 (83.1%) of them were satisfied with the availability and adequacy of medical supplies and drugs. two hundred fifty-seven participants (80.3%) were satisfied with the sufficiency and cleanliness of the labor rooms, beds, and spaces, while 185 (57.8%) of the mothers were satisfied with toilet-related services (Table 3).

**Table 3:** Structure and process related satisfaction of mothers who gave birth at public hospitals in the Somali region, Eastern Ethiopia, 2018.

Variables(items)		Structure related satisfaction (N = 320)	
		Satisfied	Unsatisfied
**Structure related satisfaction items**	Satisfaction with the number of health care provider’s	271 (84.7%)	49 (15.3%)
	Medical supply and drugs	266 (83.1%)	54 (16.9%)
	Delivery rooms and beds	257 (80.3%)	63 (19.7%)
	Satisfaction laboratory service	254 (79.4%)	66 (20.6%)
	Toilet satisfaction	185 (57.8%)	135 (42.2%)
	availability and comfort of the waiting area	246 (76.9%)	74 (23.1%)
	Satisfaction with the cost of service (N = 96)	71 (74.0%)	25 (26.0%)
**Process related Satisfaction items**	Waiting time to be seen by health care providers	271 (84.7%)	49 (15.3%)
	Satisfaction amount of time spent on examinations	251 (78.4%)	69 (21.6%)
	Satisfaction with asking permission before applying any procure	253 (79.1%)	67 (20.9%)
	Satisfaction with a health worker’s explaining labor progress in your local language	253 (79.1%)	67 (20.9%)
	Satisfaction with verbally encouraged during labor	271 (84.7%)	49 (15.3%)
	Satisfaction with competency and confidence of health care providers	266 (83.1%)	54 (16.9%)
	Satisfaction assured privacy	268 (83.8%)	5216.3%
	Satisfaction sex of the health care worker’s	251 (78.4%)	6921.6%
	Satisfaction with the care and support given to your baby	255 (79.7%)	65 (20.3%)
	Satisfaction with pain management during labor and delivery	103 (32.2%)	217 (67.8%)

#### Process-related satisfaction of the mother

The overall process-related maternal satisfaction was 76.3%. The majority of the participants, 271 (84.7%) were satisfied with the length of time they waited to be seen by the health care providers. Similarly, 251 (78.4%) of the study participants were satisfied with the amount of time health workers spent on examinations. Two hundred sixty-eight (83.8%) of the delivered mothers included in the study were satisfied with the privacy measures taken during the delivery process. Conversely, 103 (32.2%) of the study participants were satisfied with the pain management during labor and delivery (Table 3).

### Factors associated with maternal satisfaction

#### Bivariate analysis of socio-demographic and obstetric characteristics

Bivariate logistic regression analysis was done between the factors and maternal satisfaction (the outcome variable). In this analysis, those variables with a p-value of below 0.25 were considered to be candidates/covariates for the multivariable logistic regression analysis. Accordingly, eight variables namely maternal age, occupation of the mother, pregnancy status, waiting time, antenatal care, parity, duration of labor persists, and mode of delivery were moved to multivariate (Table 4).

**Table 4:** Bivariate logistic regression analysis of socio-demographic and obstetric characteristics of mothers who gave birth at public hospitals in the Somali region, Eastern Ethiopia, 2018.

Variable (n = 320)	Category	Overall Satisfaction		^a^ COR (95% ^b^ CI)	P-value
		Satisfied	Unsatisfied		
**Maternal age**	<20	47	15	1.56 (0.60,4.07)	0.35
	21–34	178	50	1.78 (0.78,4.04)	0.16
	35–49	20	10	1	
**Marital status**	Married	229	67	1.70(0.70,4.16)	0.23
	Others	16	8	1	
**Parity**	1	53	23	0.59(0.24,1.43)	0.24
	2–5	157	43	0.93(0.41,2.10)	0.87
	>5	35	9	1	
**Occupation of the**	House wife	154	54	0.59(0.28,1.21)	0.15
	Government employee	38	10	0.78(0.30,2.04)	0.62
	Others	53	11	1	
**Status of pregnancy**	Planned	225	59	3.05(1.48,6.25)	0.00
	Unplanned	20	16	1	
**Mode of delivery**	SVD	194	49	0.69(0.27,1.75)	0.44
	C/S	17	20	0.15 (0.05,0.44)	0.00
	Instrumental delivery	34	6	1	
**Length of labor**	≤12hours	157	38	1.73(1.03,2.92)	0.04
	>12hours	88	37	1	
**Waiting time**	≤15minute	230	65	2.35(1.01,5.49)	0.03
	>15minute	15	10	1	
**ANC follow up**	Yes	192	50	1.81(1.02,3.19)	0.04
	No	53	25	1	

^a^ Crude odd ratio

^b^ Confidence interval

#### Multivariate logistic regression analysis result

Eight variables were analyzed in the multivariable logistic regression model. However, only pregnancy status, waiting time, and the duration of labor persisted were found to be the independent predictors of maternal satisfaction, at a p-value of below 0.05.

Those mothers whose current pregnancy was planned were nearly four times likely to be satisfied with services than those mothers whose current pregnancy was unplanned (AOR: 3.681, 95% CI: 1.599, 8.474). Regarding the duration of labor persists; those mothers whose labor persists for 12 hours or less were nearly two times likely to be satisfied than their counterparts (AOR: 1.808, 95%CI: 1.014, 3.223).

Concerning the waiting time to be seen by the health care providers, those participants who waited 15 minutes or less were nearly three times likely to be satisfied than those who waited for more than 15 minutes (AOR: 2.781,95%CI:1.106, 6.992) (Table 5).

**Table 5:** Multi-variable logistic regression analysis for factors associated with mother’s satisfaction with delivery services in public hospitals in the Somali region, Eastern Ethiopia, 2018.

Variable (n = 320)	Category	Overall satisfaction		^a^ COR(95% ^b^ CI)	^c^AOR(95%CI)	P-value
		Satisfied	Unsatisfied			
**Status of pregnancy**	**Planned**	225	59	3.05 (1.48, 6.25)	3.68 (1.59,8.47)	0.00
	**Unplanned**	20	16	1	1	
**Length of labor**	**≤12hours**	157	38	1.73 (1.03, 2.92)	1.80 (1.01,3.22)	0.04
	**>12hours**	88	37	1	1	
**Waiting time**	**≤15minute**	230	65	2.35 (1.01, 5.49)	2.78 (1.10,6.99)	0.03
	**>15minute**	15	10	1	1	

^a^ Crude odd ratio

^b^ Confidence interval

^c^ Adjusted odd ratio

## Discussion

This was a facility-based cross-sectional study to assess maternal satisfaction and associated factors towards institutional delivery service at the public hospitals in the Somali region, Eastern Ethiopia, in 2018. Maternal satisfaction with delivery service is an important outcome measure for quality of care and provision of services. This study showed that the overall level of maternal satisfaction with delivery services was 76.6%. The structure-related and process-related satisfaction of the mothers were 76.9% and 76.3%, respectively. The duration of the labor persistence, waiting time and current pregnancy status were factors associated with the mother’s satisfaction with delivery services. The overall mother’s satisfaction with delivery service was in line with the studies conducted in Felege-hiwot referral hospital in northwest Ethiopia (74.9%), Mekelle Ethiopia (79.7%), and Debre-Markos town (80%), and Jimma University specialized hospital (77%). This is may be due to the fact that these studies were institution-based studies similar to the design used in this study [1, 5, 8, 11, 23]. However, still, a quarter of women were unsatisfied with the delivery service. Efforts should be made to bring all mothers to the level of full satisfaction, which will in turn encourage them to use other maternal and child health services like postnatal care, child vaccination, maternal vaccination, etc.

Those mothers whose current pregnancy status was planned were nearly four times more likely to be satisfied with services than those mothers whose pregnancy was unplanned. Similar studies conducted in the Omo Nada district of Jimma zone and Debre Markos town showed that mothers who planned their pregnancy were more likely to be satisfied with institutional delivery service compared to those who did not plan. This is may be due to mothers with planned pregnancies having a better awareness of attending antenatal care and other basic maternal health services. Thus, they are less likely to suffer from preventable complications, such as persistent labor, and others. This implies that the local and national programs should give a thorough emphasis on pregnancy planning through advocacy and health education. These interventions enhance the uptake of the continuum of maternal health care. It will significantly contribute to the reduction of maternal deaths from preventable childbirth-related complications [11, 24].

In terms of labor duration, mothers whose labor lasted less than 12 hours or less were nearly twice as likely to be satisfied as women whose labor lasted more than 12 hours. This finding was supported by a systematic review in Ethiopia and a study conducted in Wolaita, Southern Ethiopia. Those women who suffer from persistent labor pain are likely to be dissatisfied with the delivery service. Skilled birth attendants are key to the management of persistent labor. As a result, having a compassionate and skilled health care provider present during delivery may have an impact on maternal satisfaction and the use of continuum care [25, 26].

Regarding the waiting time to be seen by the healthcare providers, were nearly three times more likely to be satisfied compared to women who had waited more than 15 minutes. This was similar to a study conducted in Assela Hospital which showed that all the respondents’ mothers’ waiting time was to be seen by health care providers. It was also consistent with evidence from Argentina, Gambia, Bangladesh, Iran, Malawi, Nigeria, Sri Lanka, Saudi Arabia, and Uganda. This is probably due to the long waiting time hindering pregnant women from getting timely management of their child birth. These women might suffer from minor and major complications of pregnancy and childbirth. The longer waiting time might have resulted from the inadequacy of skilled providers and negligence one part of the providers. This could have a negative impact on the use of the continuum of care [12, 26–28]. Generally, the aforementioned factors result from maternal dissatisfaction with delivery service. On the other hand, the uptake of the maternal continuum of care (CoC) is a top agenda in the local and national programs to reduce maternal mortality [29]. However, maternal dissatisfaction with delivery services hinders the utilization of CoC and significantly affects the effort to reduce maternal death from preventable causes.

One of the limitations of this study was the potential response biases often present in client satisfaction studies related to social desirability. We tried to reduce this bias by interviewing mothers in a separate room with trained nurses who were not affiliated with the facilities studied. The results of this study were not triangulated with qualitative studies. Hence, we suggest future studies on maternal satisfaction should be triangulated with qualitative studies.

## Conclusions

In conclusion, three-fourth of the study participants were found to be satisfied with the delivery service provided at the public hospitals in the Somali region. Below 12 hours’ labor duration, waiting time less than 15 minutes and having a planned pregnancy were factors associated with the mother’s satisfaction. Longer waiting times, unplanned pregnancies, and suffering from persistent labor hinder maternal satisfaction with delivery service. Hence, local and national programs should enhance education and counseling on pregnancy planning. An effort should be made to obtain an adequate number of skilled and compassionate birth attendants to manage the long waiting time and suffering from persistent labor.

## Supporting information

S1 File Data set in SPSS. (SAV)
